# Folinic Acid Increases Protein Arginine Methylation in Human Endothelial Cells

**DOI:** 10.3390/nu10040404

**Published:** 2018-03-24

**Authors:** Ruben Esse, Tom Teerlink, Pieter Koolwijk, Isabel Tavares de Almeida, Henk J. Blom, Rita Castro

**Affiliations:** 1 Institute for Medicines and Pharmaceutical Sciences (iMed.UL), Faculty of Pharmacy, University of Lisbon, 1649-003 Lisbon, Portugal; italmeida@ff.ulisboa.pt (I.T.d.A.); rcastro@ff.ulisboa.pt (R.C.); 2 Department of Clinical Chemistry, Metabolic Unit, VU University Medical Center, 1081 HV Amsterdam, The Netherlands; t.teerlink@vumc.nl (T.T.); henkblom56@gmail.com (H.J.B.); 3 Department of Biochemistry, Boston University School of Medicine, Boston, MA 02118, USA; 4 Department of Physiology, VU University Medical Center, 1081 HV Amsterdam, The Netherlands; p.koolwijk@vumc.nl; 5 Laboratory for Clinical Biochemistry and Metabolism, Department of General Pediatrics, Center for Pediatrics and Adolescent Medicine University Hospital, Mathildenstrasse 1, D-79106 Freiburg, Germany; 6 Department of Biochemistry and Human Biology, Faculty of Pharmacy, University of Lisbon, 1649-003 Lisbon, Portugal; 7 Department of Nutritional Sciences, Pennsylvania State University, University Park, 16802 PA, USA; mum689@psu.edu

**Keywords:** folate, homocysteine, cellular methylation capacity, protein arginine methylation

## Abstract

Elevated plasma total homocysteine (tHcy) is associated with increased risk of cardiovascular disease, but the mechanisms underlying this association are not completely understood. Cellular hypomethylation has been suggested to be a key pathophysiologic mechanism, since *S*-adenosylhomocysteine (AdoHcy), the Hcy metabolic precursor and a potent inhibitor of methyltransferase activity, accumulates in the setting of hyperhomocysteinemia. In this study, the impact of folate and methionine on intracellular AdoHcy levels and protein arginine methylation status was studied. Human endothelial cells were incubated with increasing concentrations of folinic acid (FnA), a stable precursor of folate, with or without methionine restriction. The levels of intracellular AdoHcy and AdoMet, tHcy in the cell culture medium, and protein-incorporated methylarginines were evaluated by suitable liquid chromatography techniques. FnA supplementation, with or without methionine restriction, reduced the level of tHcy and did not affect intracellular AdoMet levels. Interestingly, FnA supplementation reduced intracellular AdoHcy levels only in cells grown under methionine restriction. Furthermore, these cells also displayed increased protein arginine methylation status. These observations suggest that folic acid supplementation may enhance cellular methylation capacity under a low methionine status. Our results lead us to hypothesize that the putative benefits of folic acid supplementation in restoring endothelial homeostasis, thus preventing atherothrombotic events, should be reevaluated in subjects under a methionine restriction diet.

## 1. Introduction

Folate intake reduces risk of neural tube defects [1] and may also protect against several other clinical conditions, including certain cancers [2,3]. Elevated levels of homocysteine (Hcy) are accepted as a risk factor/biomarker/predictor for developing cardiovascular disease [4]. However, the effect of folic acid supplementation on cardiovascular disease prevention via lowering plasma Hcy levels has been disputed. In fact, recent reviews and meta-analyzes have shown that folic acid supplementation does not reduce cardiovascular disease risk [5,6]. Moreover, it is not clear whether Hcy itself exerts an atherogenic effect and therefore is a direct causal risk factor for vascular disease [7]. Its elevation may also reflect a pathological mechanism up- or downstream in Hcy metabolism. *S*-adenosylmethionine (AdoMet) is the methyl donor in transmethylation reactions producing *S*-adenosylhomocysteine (AdoHcy). AdoHcy is further converted to Hcy through a reversible reaction in which AdoHcy synthesis, rather than its hydrolysis, is thermodynamically favored [8]. Importantly, this reaction is the only route for Hcy synthesis in humans. Several studies have shown that AdoHcy, a potent inhibitor of most methyltransferases, accumulates in the setting of hyperhomocysteinemia (HHcy) [7,9,10,11,12,13,14]. Therefore, cellular hypomethylation may underlie the vascular complications observed in HHcy [7]. In fact, several lines of evidence have shown an association between DNA hypomethylation and HHcy [9,14,15,16], but the impact of elevated Hcy and that of folate intake on other important cellular methylation reactions, including protein methylation, has been largely overlooked [17]. Part of the therapeutic potential of folate may rely on its ability to modulate methylation processes by stimulating the remethylation of Hcy to methionine, thus reducing AdoHcy concentrations and increasing AdoMet availability.

Protein arginine methylation is a post-translational modification involved in many crucial biological processes [17,18]. Protein arginine methyltransferases (PRMTs) catalyze the transfer of methyl groups from AdoMet to arginine residues in proteins. Two types of PRMTs exist. Both produce *N*^G^-monomethylarginine (MMA); however, whereas type I PRMTs form asymmetric *N*^G^,*N*^G^-dimethylarginine (ADMA), type II PRMTs form symmetric *N*^G^,*N*′^G^-dimethylarginine (SDMA). We have shown that, in human endothelial cells, AdoHcy accumulation suppresses protein arginine methylation to a higher extent than DNA methylation [19]. In a more recent study, we demonstrated that rats fed an HHcy-inducing diet (enriched in methionine and depleted in B vitamins) presented global protein arginine hypomethylation in heart and brain tissues [10]. Subsequently, we observed that mice deficient in cystathionine beta-synthase (an enzyme involved in the Hcy catabolism), displayed elevated levels of tHcy and AdoHcy and decreased protein arginine methylation status in several tissues [20]. Therefore, our observations raise the possibility that protein arginine methylation is more prone than DNA methylation to be inhibited in HHcy.

Many Hcy-lowering clinical trials with folate have been performed with the aim of reducing the risk of cardiovascular disease associated with HHcy [21]. However, these trials did not yield the anticipated cardioprotective effect [7,22]. Noteworthy, these studies have not addressed the effect of the folate interventions on the levels of other relevant metabolites, namely methionine, AdoMet and AdoHcy, and on cellular methylation processes. This lack of data prompted us to investigate the effect of folate supplementation under a low or normal methionine status on the levels of AdoMet and AdoHcy, and its impact on protein arginine methylation status in human umbilical vein endothelial cells (HUVECs). In previous studies, we used the same cell model, i.e., HUVECs, to unravel the ability of hypomethylation to promote endothelial dysfunction, a feature implicated in the vascular toxicity associated with Hcy [7,19,23,24,25,26]. Moreover, HUVECs rely exclusively on the folate-dependent remethylation pathway to clear intracellular Hcy [8], which confers an additional advantage in using this model to study the effect of folinic acid supplementation. 

## 2. Materials and Methods

### 2.1. Materials

Hepes, methionine (Met), phenylmethanesulfonyl fluoride, and 5-formyltetrahydrofolate (folinic acid (FnA)) were obtained from Sigma-Aldrich (St Louis, MO, USA). l-glutamine was purchased from Biochrom-AG (Berlin, Germany). Newborn calf bovine serum and endothelial cell growth factor were from Roche (Mannheim, Germany) and collagenase, M199 basal culture medium (with Earle’s balanced salt solution and Hepes), and Hank’s balanced salt solution were from Promocell (Heidelberg, Germany).

### 2.2. Cell Culture

HUVECs were cultivated as previously described [19], except that a custom-made basal M199 medium was used without Met and folate (Promocell, Heidelberg, Germany). FnA and Met were added to the incubation medium to the final concentrations of 2.5 nmol L^−1^ and 10 µmol L^−1^, respectively, to ensure cell viability and growth. HUVECs were cultivated in this medium in gelatin-coated 6-well plates until reaching 75–90% confluence after 2 or 3 passages. The experiment was then initiated by replacing the incubation medium with fresh medium supplemented with Met (10 or 100 µmol L^−1^) and FnA (0, 10, 50, or 500 nmol L^−1^). After 48 h, aliquots of the incubation medium were collected. Whole cell lysates were prepared by incubation in ice-cold lysis buffer (Cell Signaling Technology, Frankfurt am Main, Germany) with 1 mmol L^−1^ phenylmethanesulfonyl fluoride for 15 min and then centrifuged to remove cellular debris. Total protein was measured by the Bicinchoninic Acid Protein Assay Kit (Pierce, Rockford, IL, USA) using bovine serum albumin as the standard. Lysates were stored at −80 °C until further use.

### 2.3. Determination of Total Hcy (tHcy) in Medium and of Intracellular AdoMet and AdoHcy

Total Hcy (free plus protein-bound plus disulfide) levels in culture medium were measured by HPLC coupled with fluorimetric detection, as previously described [24]. For intracellular AdoMet and AdoHcy determination, whole cell lysates were deproteinized with equal volumes of 10% perchloric acid, centrifuged at 4 °C, 16,000 *g*, for 2 min, and the obtained supernatant was analyzed by stable-isotope dilution liquid chromatography-tandem mass spectrometry (LC-MS/MS), as previously described [19].

### 2.4. Evaluation of Protein Arginine Methylation Status in Cells

Protein arginine methylation status was evaluated as the ratios of each methylarginine to total arginine content in proteins from cell lysates, as previously described [19].

### 2.5. Statistical Analysis

All experiments were performed with cells from individual donors (*n* ranged from 5 to 9). In the plotted data, each box represents the interquartile range, the horizontal line bisecting it is the median, and whiskers are the minimum and maximum values. Statistical significance of the effect of the increasing concentrations of FnA was determined using repeated measures ANOVA with Tukey’s post-hoc analysis and was accepted at *p* < 0.05 (versus 0 nmol L^−1^ FnA). Statistical significance of the effect of Met concentration in the incubation medium was tested using a Student’s paired *t*-test and was accepted at *p* < 0.05 (100 µmol L^−1^ Met versus 0 µmol L^−1^ Met). 

## 3. Results

### 3.1. High Methionine Availability Offsets the Effect of Folinic Acid on Homocysteine Export from Endothelial Cells

**Figure 1:**
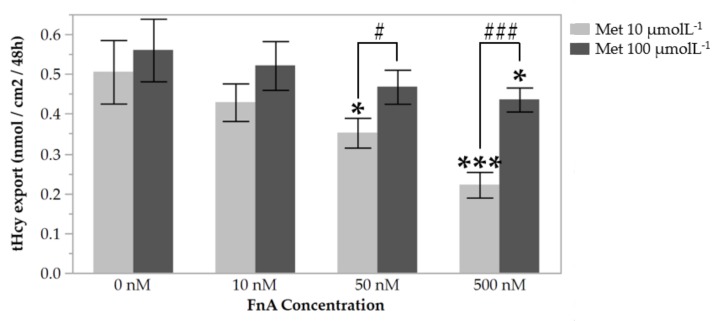
Export of total homocysteine (tHcy) from HUVECs during 48 h of incubation in the presence of increasing concentrations of folinic acid (FnA), and with methionine (Met) at 10 µmol L^−1^ or 100 µmol L^−1^. Values are mean ± SE and represent 5–9 independent experiments with HUVECs from individual donors. Statistical significance of the effect of the increasing concentrations of FnA was determined using one-way ANOVA with Tukey’s post-hoc analysis. * and *** denote *p* values < 0.05 and <0.001, respectively. Statistical significance of the effect of Met concentration was determined using a Student’s paired *t*-test. # and ### denote *p* values < 0.05 and <0.001, respectively.

Incubation of HUVECs with FnA, a stable precursor of folate, in the presence of either dose of Met tested elicited a dose-dependent decrease in the concentration of tHcy in the cell culture medium (Figure 1). This effect has been described before and is attributed to the enhancement of the folate-dependent Hcy remethylation pathway [27,28]. Cells incubated with 100 µmol L^−1^ Met produced higher amounts of tHcy than those incubated with 10 µmol L^−1^ Met. Therefore, the effect of Met on tHcy export is opposed to that of FnA.

### 3.2. Folinic Acid Decreases Cellular Methylation Capacity in Endothelial Cells under Low Methionine Availability

**Figure 2:**
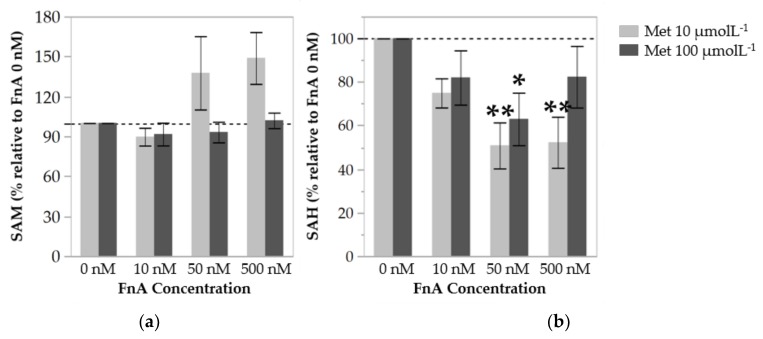
Relative intracellular concentrations of *S*-adenosylmethionine (AdoMet) (**a**) and *S*-adenosylhomocysteine (AdoHcy) (**b**) in HUVECs after 48 h of incubation with increasing concentrations of folinic acid (FnA), and with methionine (Met) at 10 µmol L^−1^ or 100 µmol L^−1^. Values are mean ± SE, are relative to cells incubated in the absence of FnA, and represent 5–9 independent experiments with HUVECs from individual donors. Statistical significance of the effect of the increasing concentrations of FnA was determined using one-way ANOVA with Tukey’s post-hoc analysis. * and ** denote *p* values < 0.05 and <0.01, respectively.

Because the levels of intracellular AdoMet and AdoHcy displayed variation among the different cell lines used in this study, these results are expressed as percentage of the cell control levels (in the absence of FnA). AdoMet levels were not affected by supplementation of FnA at either dose of Met tested (Figure 2a). However, AdoHcy levels in cells supplemented with FnA 50 nmol L^−1^ and 500 nmol L^−1^ were approximately two-fold lower than in cells incubated in the absence of FnA, but only at the lowest dose of Met (Figure 2b). These observations suggest that FnA supplementation does decrease cellular methylation capacity, but only in cells under Met restriction.

### 3.3. Folinic Acid Lowers Protein Arginine Methylation in Endothelial Cells under Low Methionine Availability

**Figure 3:**
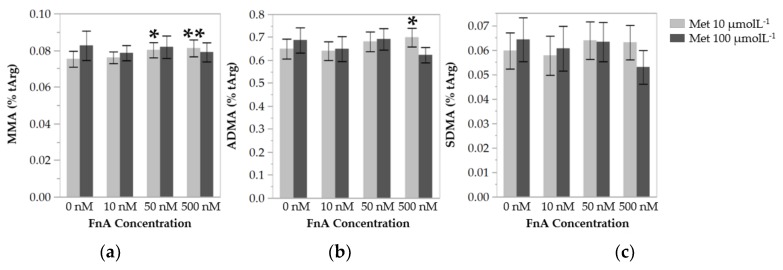
Concentrations of protein-incorporated *N*^G^-monomethylarginine (MMA) (**a**), asymmetric *N*^G^,*N*^G^-dimethylarginine (ADMA) (**b**), and symmetric *N*^G^,*N*′^G^-dimethylarginine (SDMA) (**c**) in HUVECs after 48 h of incubation with increasing concentrations of folinic acid (FnA), with methionine (Met) at 10 µmol L^−1^ or 100 µmol L^−1^. Values are mean ± SE, are expressed as fraction of total arginine content, and represent 5–9 independent experiments with HUVECs from individual donors. Statistical significance of the effect of the increasing concentrations of FnA was determined using one-way ANOVA with Tukey’s post-hoc analysis. * and ** denote *p* values < 0.05 and <0.01, respectively.

As a functional read-out of methylation capacity, we determined the levels of protein-incorporated MMA, ADMA, and SDMA (Figure 3). At the low dose of Met, cells incubated in the presence of 50 nmol L^−1^ and 500 nmol L^−1^ of FnA displayed, on average, 6.5% and 7.9% higher levels of protein-incorporated MMA, respectively, than cells incubated in the absence of FnA. The levels of protein-incorporated ADMA in cells exposed to the highest dose of FnA were on average 7.7% higher than in cells incubated without FnA. FnA had no significant effect on the levels of protein-incorporated SDMA, which may be related to the fact that SDMA levels were too low for any significant alteration to be detected. At the high dose of Met, FnA supplementation had no effect on the levels of protein-incorporated methylarginines.

## 4. Discussion

The lowering effect of folate on circulating Hcy levels in humans has been thoroughly documented [21]; interestingly, oral administration of FnA has been proven to be comparably efficacious [29]. In fact, FnA is converted to 5-methyltetrahydrofolate (5-mTHF), the active form of folate, through the enzymatic activity of 5,10-methenyltetrahydrofolate synthetase and methylenetetrahydrofolate reductase (MTHFR) [30]. In this study, we observed a dose-dependent decrease in extracellular tHcy concentrations in HUVECs exposed to FnA, probably reflecting an increase of biochemical clearance of Hcy by remethylation. The observed increase in tHcy in the medium of cells exposed to 100 µmol L^−1^ Met, compared with those exposed to 10 µmol L^−1^ Met, suggests that increased Met availability increases the production of Hcy via AdoMet-dependent methyl transfer reactions. Furthermore, since AdoMet is an allosterical inhibitor of MTHFR [31,32], increased AdoMet concentration due to excessive Met may further constrain the folate-dependent remethylation pathway, resulting in Hcy build up and export to the cell culture medium. Thus, increased availability of Met partially abolishes the Hcy-lowering effect of FnA in HUVECs.

At present, it is not clear whether increased availability of folate affects the levels of the Hcy precursors AdoMet and AdoHcy, and hence alter cellular methylation processes. One study has shown that plasma folate is inversely correlated with tHcy level, but not with that of AdoHcy [33]. Moreover, B-vitamin supplementation in older people, albeit successful in lowering plasma tHcy, had no effect on AdoMet and AdoHcy concentrations [34]. In these studies, AdoMet and AdoHcy levels were assessed in plasma. If reduced cellular methylation capacity is indeed detrimental to vascular health and homeostasis, we reason that the intracellular AdoMet and AdoHcy concentrations are better indicators of cellular methylation capacity than the levels of the circulating metabolites. In our hands, the intracellular levels of AdoMet in cultured endothelial cells were not affected by FnA supplementation, which is in line with our previous data showing that dietary manipulation of Met and B vitamins in Wistar rats does not alter AdoMet levels in different tissues [10]. Taken together, these findings suggest that the intracellular AdoMet concentration is tightly regulated and is not sensitive to Met and FnA status. Interestingly, the levels of AdoHcy were found to be decreased upon FnA supplementation, but only in cells under Met restriction. We speculate that, under a high availability of Met and hence of labile methyl groups, Hcy is produced at a rate such that the Hcy remethylation pathway, even if enhanced by folate supplementation, is ineffective in clearing Hcy. The allosteric inhibitory effect of AdoMet and AdoHcy on MTHFR may be potentiated by excessive Met and thus contribute to the lack of efficacy of the remethylation pathway. We conclude that FnA is effective in promoting a decrease of AdoHcy levels, leading to an increase of cellular methylation capacity, but only in the presence of low Met availability.

Protein arginine methylation was globally increased in cells exposed to FnA. This effect was modest, yet statistically significant at the highest dose of FnA for both MMA and ADMA, and, noteworthy, only in cells subject to Met restriction. These results suggest that, in the context of low Met availability, FnA may modulate global protein arginine methylation, albeit moderately, by lowering intracellular AdoHcy levels.

The results disclosed here show that, at low Met concentrations, administration of FnA lowers not only the level of intracellular AdoHcy, the endogenous methyltransferase inhibitor, but also that of Hcy, most likely by enhancement of its remethylation to Met. In this study, we also present evidence that FnA increases the degree of protein arginine methylation, putatively by preventing the inhibition of PRMTs by AdoHcy. It should be taken into account that the effect of FnA on AdoHcy and on protein arginine methylation status was only observed at the low dose of Met used. Therefore, if our observations translated to the in vivo situation, they could suggest that folate intervention may only offer a therapeutic value if combined with dietary methionine restriction.
